# Epitaxial Growth of Large-Scale α-Phase Antimonene

**DOI:** 10.1021/acs.nanolett.4c03277

**Published:** 2024-09-24

**Authors:** Tomasz Jaroch, Lucyna Żurawek-Wyczesany, Agnieszka Stȩpniak-Dybala, Mariusz Krawiec, Marek Kopciuszyński, Piotr Dróżdż, Mariusz Gołȩbiowski, Ryszard Zdyb

**Affiliations:** Institute of Physics, Maria Curie-Sklodowska University, 20-031 Lublin, Poland

**Keywords:** α phase antimonene, large scale growth, LEEM, ARPES, STM, DFT

## Abstract

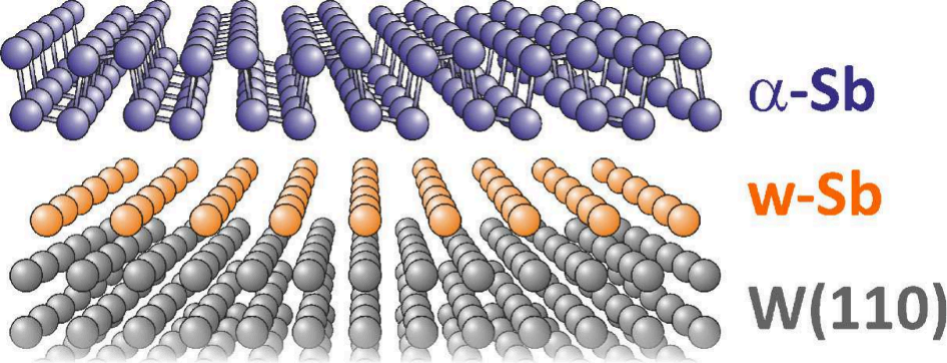

Two-dimensional materials composed of elements from the 15th group of the periodic table remain largely unexplored. The primary challenge in advancing this research is the lack of large-scale layers that would facilitate extensive studies using laterally averaging techniques and enable functionalization for the fabrication of novel electronic, optoelectronic, and spintronic devices. In this report, we present a method for synthesizing large-scale antimonene layers, on the order of cm^2^. By employing molecular beam epitaxy, we successfully grow a monolayer film of α-phase antimonene on a W(110) surface passivated with a single-atom-thick layer of Sb atoms. The formation of α phase antimonene is confirmed through scanning tunneling microscopy and low-energy electron diffraction measurements. The isolated nature of the α-phase is further evidenced in the electronic structure, with linearly dispersed bands observed through angle-resolved photoelectron spectroscopy and supported by ab initio calculations.

In recent years, there has been a tremendous surge of interest in the search for novel two-dimensional (2D) materials. Atomically thin layers exhibit numerous physical and chemical properties that are absent in their three-dimensional counterparts. These materials are anticipated to be the building blocks of future advanced electronics and other functional devices, such as detectors, catalysts, and systems for energy harvesting and storage, among others.^1,2^ However, many properties of 2D materials are currently known only through theoretical calculations, and experimental validation remains challenging due to the small sizes of 2D samples, which are typically below hundreds of micrometers.^1,3^

The next steps in the development of 2D materials involve the experimental verification of theoretically predicted properties, further investigation using lateral averaging techniques, and eventually, the production of functional devices. However, the fabrication of novel devices will heavily depend on the availability of low-cost, reliable sources of large-area 2D materials. Consequently, the fabrication of single-crystalline, high-quality 2D materials on a scale of centimeters or more has become a critical focus of current research, both theoretical and experimental.^3−8^

Currently, aside from the well-developed technology for producing large-scale graphene sheets,^9−11^ the fabrication of other two-dimensional (2D) van der Waals materials at centimeter scale or larger is still in its early stages. The top-down approach generally yields only small-sized 2D flakes, which have nonuniform thicknesses.^1^ Among bottom-up methods, chemical vapor deposition (CVD) and its derivatives are commonly used to prepare larger-scale samples, although in most cases, the resulting sizes are still smaller than a millimeter.^3,12^ There have been a few exceptions in recent years where large-scale synthesis via CVD has been reported. Besides graphene, these are hexagonal boron nitride (h-BN)^1,12−14^ and transition metal dichalcogenides (TMDs).^4,15,16^ In the case of TMDs, a new method for synthesizing wafer-scale samples has also been proposed.^4,7,8^

Another well-known bottom-up approach for fabricating 2D materials is molecular beam epitaxy (MBE). This method is particularly effective in producing uniform and crystalline layers with precisely controlled thicknesses, often down to a single monolayer. These capabilities are crucial for both basic research and technological applications of 2D materials. High-quality 2D films minimize defects, such as grain boundaries, which introduce additional channels for charge scattering reducing in this way the mobility of charge carriers. An even more significant advantage of MBE is its precise control over the number of layers, which is critical because the properties of 2D semiconductors strongly depend on layer count.^17,18^ This includes the character of a band gap (whether direct or indirect), its value, or even transition to a metallic state^17,18^—all of which are key factors that determine the performance and viability of electronic devices.

To date, with one exception,^19^ there have been no reports of centimeter-scale or larger van der Waals-type 2D materials being grown using the MBE technique. However, it is worth noting that recent reports have described approximately cm^2^-sized layers for non-van der Waals 2D materials, including a heterostructure of planar and buckled phases of silicene^20−22^ and a peculiar form of antimonene.^23^

Among the broad family of 2D elemental systems, those composed of elements from the 15th group of the periodic table—such as phosphorene, arsenene, antimonene, and bismuthene—have garnered significant attention. These materials are believed to exhibit high charge carrier mobility and possess a direct band gap, which can also be tuned. Among this group, antimonene stands out as particularly interesting.

The stability of single-layer antimony was theoretically investigated by Zhang et al.^18^ Shortly thereafter, two of the nine predicted phases, specifically α (rectangular) and β (hexagonal), Figure 1, were successfully synthesized through various methods. These include top-down approaches such as mechanical^24,25^ and liquid-phase^26^ exfoliation, as well as bottom-up techniques such as physical vapor deposition^27^ and molecular beam epitaxy.^28−31^ In all instances, separated nanoflakes or submillimeter islands were reported. However, very recently, it was demonstrated that large-scale β phase antimonene can be grown on a copper oxide substrate.^19^

**Figure 1:**
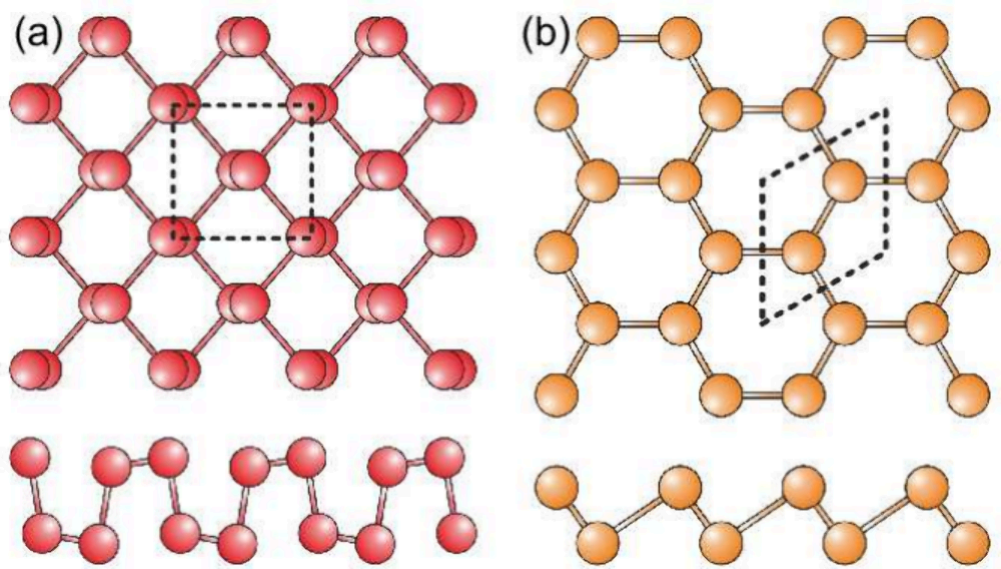
Structural models of (a) α and (b) β phases of antimonene; dashed lines indicate the unit cells.

Further studies of antimonene have uncovered numerous intriguing properties with potential applications. Its semiconducting nature, combined with the ability to tune the energy gap by varying the number of layers^17,18^ or applying strain,^32^ makes antimonene an excellent candidate for optoelectronic applications, including photovoltaics^33,34^ and ultrafast optoelectronics.^35,36^ Additionally, antimonene exhibits significant flexibility, which can drive quantum phase transitions and enable the quantum spin Hall effect,^37^ as well as the emergence of unpinned Dirac states.^38^ Interestingly, theoretical calculations suggest that ferromagnetic or antiferromagnetic order can be induced in antimonene through the application of strain, adsorption of transition-metal atoms, or proximity effects.^32,39−42^ It has also been demonstrated that antimonene remains stable under ambient conditions, a critical factor for future applications.^24,29^ These exceptional properties, along with others such as electrocatalysis, energy storage, and potential biomedical applications like cancer therapy and biosensing, could form the foundation for a new generation of devices, including electronic, spintronic, valleytronic, optoelectronic, and thermoelectronic technologies.^2,43^ Therefore, the preparation of high-quality, large-scale antimonene layers is highly anticipated.

In this study, we demonstrate the synthesis of large-scale α-phase antimonene, with areas on the order of cm^2^, using the MBE method. We investigate the growth, layer morphology, and crystallographic structure in situ using low-energy electron microscopy/diffraction (LEEM/LEED) and scanning tunneling microscopy (STM). The electronic structure of antimonene is determined through angle-resolved photoemission spectroscopy (ARPES) and density functional theory (DFT) calculations. Our results confirm the epitaxial growth of a homogeneous, high-quality single layer of α-phase antimonene, as well as its stability at room temperature.

The growth of α-phase antimonene was performed on a monocrystalline W(110) surface covered with a single-atom-thick antimony film.^23^ This additional layer of antimony (w-Sb) extends over a large area, on the order of cm^2^, and passivates the W(110) surface. The w-Sb film is relatively strongly bonded to the tungsten substrate and replicates the W(110) surface morphology, which consists of numerous monatomic steps. As shown by STM profiles in ref (23), the resulting surface remains very flat. The w-Sb film is formed after the deposition of 0.5 monolayer (ML) of Sb, where 1 ML corresponds to a density of Sb atoms in a complete single layer of α-phase antimonene, which is 20 × 10^14^ atoms/cm^2^.

Two different growth procedures were found to produce high-quality single layers of α-antimonene. In the first method, the antimonene layer was grown on a substrate maintained at a constant temperature of 390 K (Figures 2a–e). In the second method, the substrate temperature was increased from 350 K to 430 K after the deposition of 0.1 ML of antimony (see Figures 2f–j).

**Figure 2:**
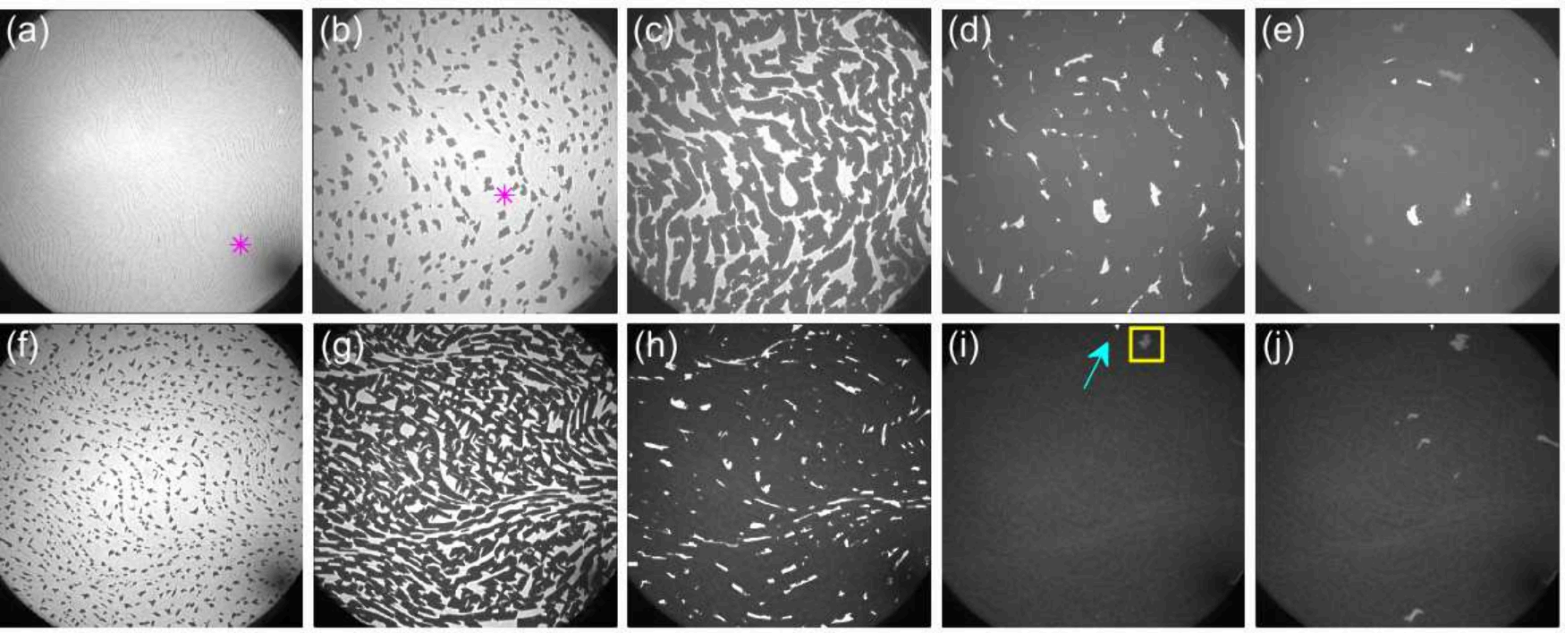
Epitaxial growth of the α-Sb layer for both one-step (upper row) and two-step (lower row) procedures. LEEM images before (a) and during Sb deposition (b–j) on the w-Sb/W(110) substrate: 0.11 ML (panel (b)), 0.56 ML (panel (c)), 0.86 ML (panel (d)), and 0.92 ML (panel (e)) for the one-step procedure, and 0.09 ML (panel (f)), 0.55 ML (panel (g)), 0.86 ML (panel (h)), 1.00 ML (panel (i)), and 1.01 ML (panel (j)) for the two-step procedure. The bright areas represent w-Sb, the dark areas indicate the first ML of α-antimonene, and the gray areas in panels (e), (i), and (j) denote islands of the second ML. The magenta asterisk in panels (a) and (b) highlights the same surface region, with minor image shift attributed to thermal drift of the sample. The upper and lower rows of images were recorded from different sample regions. The cyan arrow (yellow square) in panel (i) indicates w-Sb (2 ML α-Sb). All images were taken with an energy of *E* = 3.5 eV and a field of view (FoV) of 10 μm. The growth temperatures were *T* = 390 K for the upper row and *T* = 350 K (panel (f)) and *T* = 430 K (panels (g)–(j)) for the lower row. Full movies of the antimonene growth can be found in Movies S1A and S1B in the Supporting Information (SI).

According to the constant temperature procedure, the formation of α-antimonene is restricted to a narrow temperature range of ∼390 ± 10 K and begins immediately after the completion of the w-Sb layer. Initially, the growth of α-Sb is influenced by the substrate morphology. Figure 2a shows a LEEM image of the W(110) surface before Sb deposition, where thin dark lines denote the monatomic steps of the tungsten substrate that separate the (110)-oriented terraces. These steps remain clearly visible after the formation of the w-Sb layer and during the subsequent growth of α-Sb, as shown between the islands in Figure 2b.

The nucleation of the α antimonene layer begins at the step edges. Initially, antimonene islands grow both along and across the terraces but typically do not extend beyond the step edge where they nucleate. Instead, they smoothly follow the edge and fill out the terraces (Figure 2b). At this stage of Sb growth, the monatomic steps of the substrate act as a barrier, leading to anisotropic growth of the α-phase islands. The growth of antimonene is thus influenced by the shape of the substrate terraces.

As Sb coverage increases and antimonene islands reach the opposite edge of a terrace, the potential barrier on the other side of the terrace is sufficiently low for Sb to cross it. When coverage reaches ∼90%–100%, depending on the substrate temperature, morphology, and cleanliness, nucleation of the second layer begins on top of the first one (Figure 2e).

In the second procedure, the growth is performed at 350 K up to 0.1 ML. This process differs slightly from the deposition at 390 K: the antimonene islands are smaller and their density is higher. The lower substrate temperature results in a shorter diffusion length, causing nucleation of islands not only at the step edges but also on the substrate terraces. Subsequently, the substrate temperature is increased to 430 K, and growth continues until the antimonene layer is completed. The higher density of islands after the initial low-temperature deposition provides numerous nucleation centers for the high-temperature growth. Consequently, the layer becomes uniformly covered at 1 ML (Figure 2i). However, some very small areas of w-Sb (indicated by the cyan arrow) and islands of the second layer (within the yellow square) are still present.

Movies S1A and S1B, along with Movie S2 in the Supporting Information (SI), present the growth of α-antimonene with fields of view of 10 and 50 μm, respectively. It is important to note that the antimonene layer uniformly covers the surface across the studied area, which spans several millimeters in diameter.

Substrate temperatures that are too high during Sb deposition do not promote the formation of a continuous layer. Instead, the extended diffusion length leads to the formation of large, isolated α-Sb islands, which then serve as substrates for the growth of subsequent Sb layers (see Movie S3 in the SI). Conversely, excessively low temperatures reduce the diffusion length, resulting in a higher density of nucleation sites and consequently smaller crystallites. This leads to a greater density of grain boundaries and domain walls. Additionally, nucleation of subsequent layers begins significantly earlier, before the first layer is fully completed (see Movie S4 in the SI).

It is important to note that the antimonene layer maintains its morphology and does not break into separate islands, and it preserves its crystallographic structure after cooling to room temperature. No phase transition from α to β phases occurs, as observed in ref (44), and the lattice constants remain unchanged. Conversely, the single α-antimonene layer remains stable up to ∼450 ± 10 K. Above this temperature, Sb desorption begins, and the layer is completely removed at ∼500 ± 10 K, leaving the W(110) surface covered by the w-Sb film. This relatively low desorption temperature suggests a weak bonding of the antimonene layer to the underlying w-Sb/W(110) substrate.

The LEED pattern of the α antimonene layer displays numerous sharp diffraction spots that are symmetric with respect to the [11̅0] and [001] directions of the W(110) substrate (Figure 3a). These diffraction spots correspond to both the antimonene layer and the underlying w-Sb layer. Two sets of arrows (solid blue and dashed magenta) indicate the basic vectors of the two reciprocal unit cells of the centered rectangular structure. The resulting two mirror domains form an angle of 94° ± 2° between their basic vectors.

**Figure 3:**
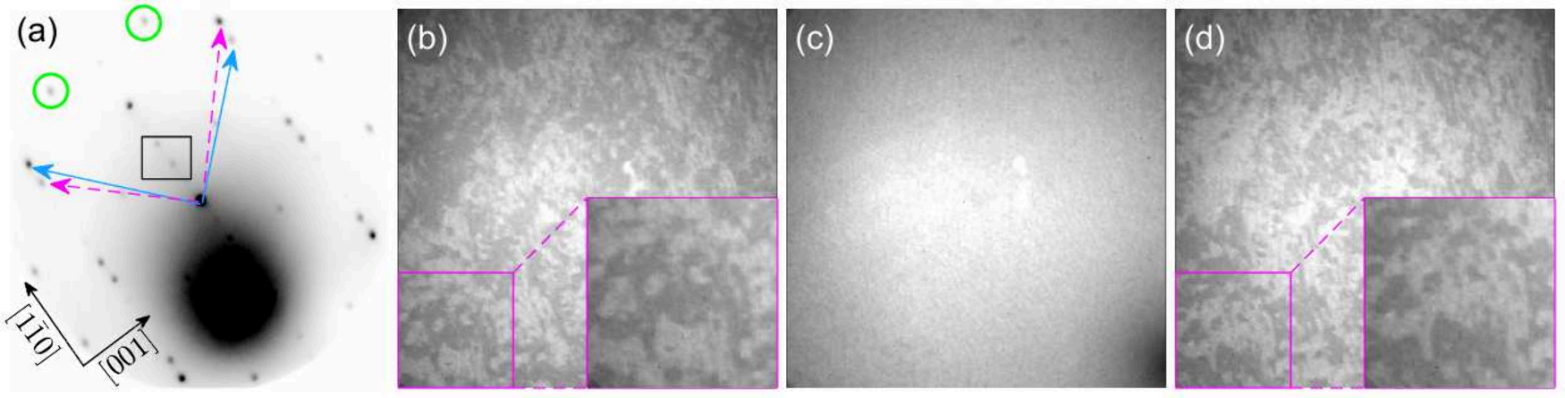
(a) LEED pattern of α-Sb with inverted contrast (dark areas indicate higher intensity). Blue and magenta arrows denote the reciprocal unit cell vectors of the two mirror domains. Green circles highlight exemplary diffraction spots of the (1 × 2) superstructure. The spots corresponding to the w-Sb layer are marked within the black rectangle. The large black area represents secondary electrons. The characteristic directions of the W(110) substrate are indicated in the lower left corner. *E* = 40 eV. (b)–(d) LEEM images taken with the electron beam tilted in opposite directions (panels (b) and (d)) and at normal incidence (panel (c)). The sample region in the bottom left corner is enlarged and shown on the right side of the images in panels (b) and (d). Size of LEEM images: 3.5 μm × 3.5 μm, *E* = 22 eV.

Both domains exhibit slightly distorted rectangular unit cells with an angle of 94° ± 2° and lattice constants of *a* = 4.32 Å and *b* = 4.69 Å (± 0.08 Å). A similar two-domain structure has been reported for the α-phase antimonene grown on WTe_2_^45^ and for several monolayer-thick films of Bi(110).^46^ Additionally, the α-Sb layer exhibits a (1 × 2) superstructure, with two of the corresponding diffraction spots highlighted by green circles in Figure 3a.

In addition to the diffraction spots associated with α-Sb, there are also considerably weaker spots originating from the underlying Sb layer that passivates the tungsten substrate.^23^ Two of these weaker spots are indicated within the black rectangle in Figure 3a.

The two-domain structure of the α-Sb layer is also evident in the LEEM images taken with a slightly tilted incident electron beam (Figures 3b and 3d). Under these experimental conditions, the tilt breaks the symmetry of the experiment, effectively mimicking dark-field imaging. Consequently, the domains appear as alternating bright and dark areas. Reversing the tilt of the electron beam switches the contrast of the domains: bright regions become dark and vice versa. At normal incidence (no tilt), Figure 3c, no contrast between the domains is observed. The lateral size of the domains ranges from tens to hundreds of nanometers. The evolution of the two-domain structure during the growth of the α-Sb layer is further illustrated in Movie S5 in the SI.

Since the antimonene layer is composed of mirror domains, the formation of grain boundaries between these domains is inevitable. Another potential source of structural defects arises from the merging of islands to form a continuous layer, including nucleation at substrate step edges. Structural defects, such as grain boundaries, are known to impact the transport properties of the layer by reducing charge carrier lifetime and, consequently, decreasing their mobility. To enhance the performance of the material for potential applications, it is crucial to minimize the density of grain boundaries. One approach to achieve this is by reducing the density of substrate defects, e.g., surface steps.

STM measurements confirm the formation of the α-phase antimonene (Figure 4). The measured distances between Sb atoms of 4.2 and 4.7 Å (±0.1 Å) along both directions of the unit cell are consistent with the results obtained from LEED measurements within the error margin. Additionally, the angle between the basic vectors, measured as 93° ± 2° via fast Fourier transform (FFT) analysis (see inset in Figure 4a) corroborates the diffraction results.

**Figure 4:**
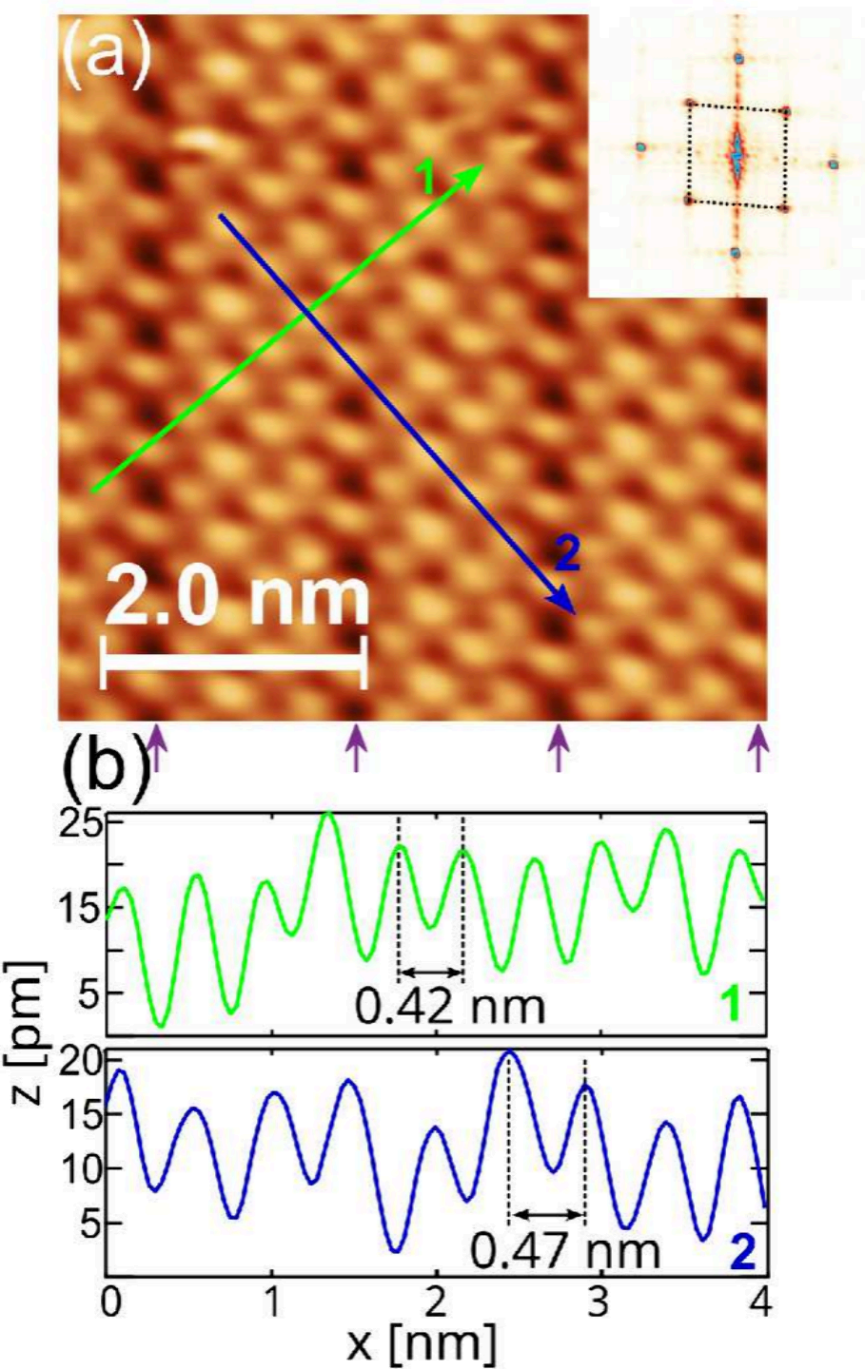
(a) STM image of α-Sb grown on the w-Sb/W(110) surface. The profiles in panel (b) were taken along the green and blue lines. The inset in panel (a) presents the FFT pattern obtained from the STM image. The arrows below the STM image highlight the Sb atomic rows with a darker interatomic background. Image size: 5.5 nm × 5.5 nm, *U*= −400 mV, *I* = 50 pA. STM measurements were performed at *T* = 4.5 K.

The profiles shown in Figure 4b indicate that the α-Sb layer is atomically flat, with a corrugation of ∼10 pm. The vertical atomic rows with a darker interatomic background, indicated by the arrows below the image, are attributed to the underlying w-Sb layer. These rows reveal the internal structure of the w-Sb layer^23^ and align with the [001] direction of the tungsten substrate. Using this reference, we determined that the unit cell of the α-Sb layer is rotated by ±(42° ± 2°), relative to the [001] direction of W(110), consistent with the electron diffraction results.

The growth of large-scale α-antimonene layers enables the use of laterally averaging techniques, including photoemission measurements. Figures 5a and 5b present ARPES maps taken along one side of the α-Sb unit cell (ΓY) (Figure 5a) and in the perpendicular direction at *k*_*y*_ = 0.53 Å^–1^ (Figure 5b), corresponding to the green dotted line in Figure 5c. In addition to the intense, wide, parabolic-like bands from the w-Sb/W(110) substrate, there are also bands with linear dispersion crossing the Fermi level. These narrow linear bands extend more than 1.0 eV below the Fermi level. The constant energy cut shown in Figure 5c reveals that the linearly dispersed bands form an asymmetric Dirac cone, which appears as a rounded rectangle rather than a circle. This distortion arises from the lower symmetry of the system compared to, for example, graphene. However, it is important to note that, even in graphene, the Dirac cone can exhibit distortions due to anisotropic substrate potentials (see ref (47) and references therein).

**Figure 5:**
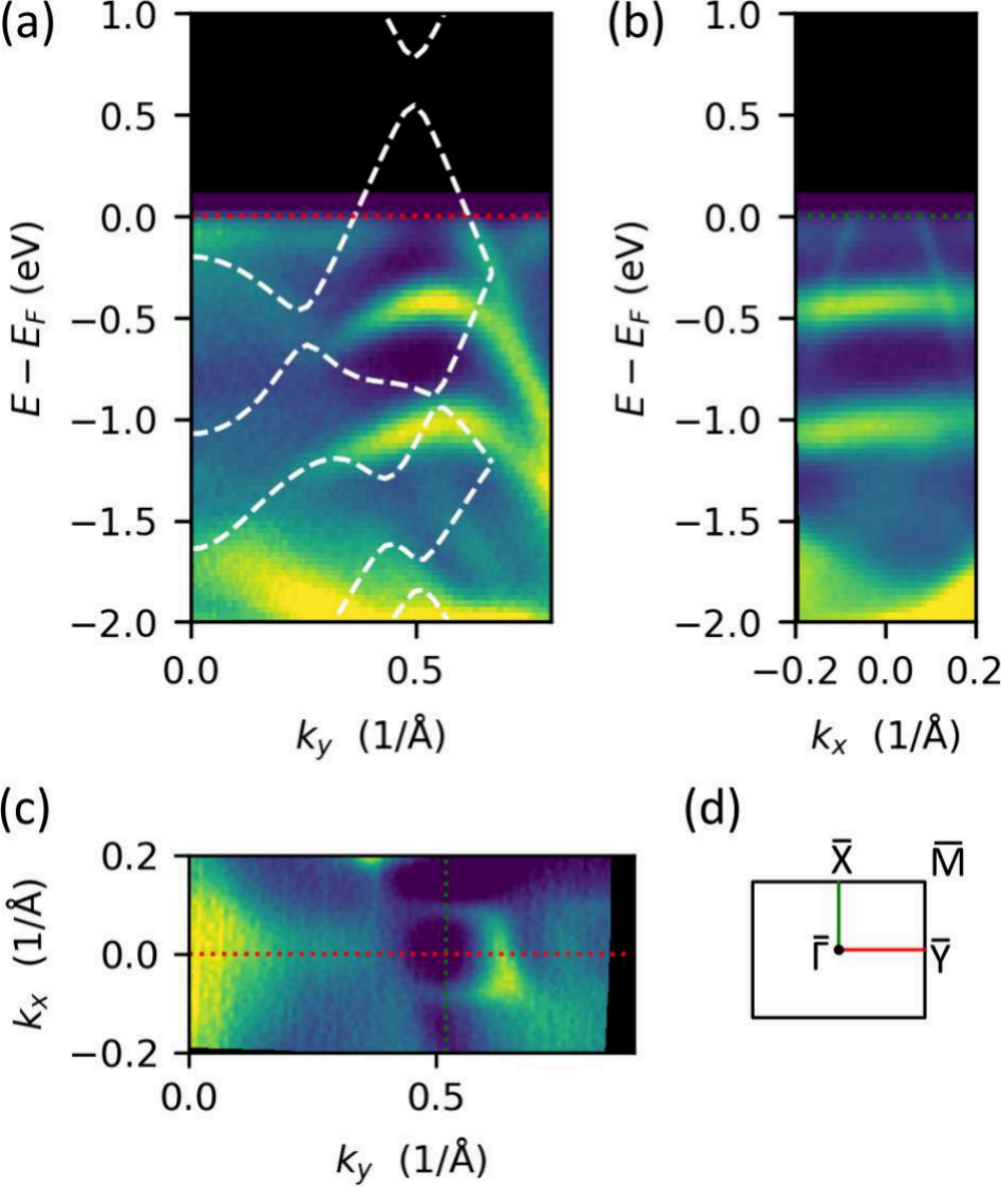
(a, b) ARPES maps of α-Sb measured along two perpendicular directions, shown as dotted lines in panel (c). Zero energy denotes the Fermi level. (c) Constant energy cut at the Fermi level. The white dashed lines in panel (a) present results of DFT calculations of electronic structure along the ΓY direction. (d) The Brillouin zone of α-Sb.

To understand our experimental results, we performed first-principles DFT calculations for two scenarios: free-standing and strained antimonene films. In the first case, the α-Sb layer was modeled without any restrictions. The resulting unit cell had lattice constants of *a* = 4.36 Å and *b* = 4.74 Å, which are consistent with other studies^17,45,48^ and closely match the values obtained from LEED and STM experiments. In the second case, the lattice constants *a* = 4.32 Å and *b* = 4.69 Å, as determined experimentally, were fixed during the calculations.

Based on both models, the band structure of the α-antimonene layer was determined. The resulting electronic structures are very similar: in both cases, linear bands appear along the ΓY direction, with a direct band gap at the Fermi level.

The electronic structure calculated for the experimentally determined unit cell parameters is overlaid on the ARPES map with white dashed lines in Figure 5a. To match the experimental results, the theoretical band structure was shifted upward by 0.7 eV. This shift accounts for the absence of the w-Sb/W(110) substrate in the calculations. Additionally, the lack of the supporting layer in the theoretical model explains the absence of the bands associated with the w-Sb/W(110) substrate, which are visible in the ARPES data.

It is noteworthy that reported lattice constants for the α-Sb single layer vary across different studies.^44,49,50^ This variation suggests that the choice of substrate with different crystallographic properties can influence the observed lattice constants. These differences indicate the flexibility of the α-phase antimonene and its potential for strain engineering. Indeed, theoretical predictions suggest that this phase of antimonene can accommodate very large strains, up to 30%.^17,48^

In conclusion, we have successfully prepared large-area, of the order of cm^2^, α-phase antimonene layers. Diffraction and microscopy experiments confirm the growth of a well-ordered antimonene film, which is exceptionally flat and reproduces the morphology of the underlying tungsten substrate. These results suggest that α-phase antimonene could be produced on an even larger scale, provided the tungsten surface is atomically clean and maintained at an elevated temperature during antimony deposition.

## Methods

### Experimental Section

All experiments were conducted in three separate ultrahigh vacuum (UHV) systems, each operating at a base pressure in the mid 10^–11^ mbar range. These UHV systems are equipped with antimony effusion cells. The crystallographic structure and morphology of the antimonene layer were characterized using low-energy electron microscopy (LEEM) and scanning tunneling microscopy (STM). The electronic structure was examined with angle-resolved photoelectron spectroscopy (ARPES).

W(110) was used as the substrate for antimonene growth. Prior to Sb deposition, the W(110) substrate was cleaned using a standard procedure: annealing in an oxygen atmosphere (at 10^–7^ mbar) at 1400 K, followed by flashing to ∼2200 K to remove residual oxygen. Antimony was deposited from a resistively heated effusion cell at a rate corresponding to 1 layer of antimonene per 5 min.

The growth of antimony on the W(110) substrate begins with the formation of a single-atom-thick Sb layer (w-Sb),^23^ which has a complex crystallographic structure and is relatively strongly bonded to the W(110) substrate. On top of this w-Sb layer, the α-phase antimonene was subsequently grown.

For STM and ARPES measurements, the sample was initially prepared in the LEEM apparatus, where the w-Sb film was covered with an additional six layers of Sb. After this, the sample was transferred from the UHV environment to the STM or ARPES system. A mild annealing at ∼500 K under UHV conditions was then applied to remove antimonene layers.

It is known that antimonene is resistant to ambient conditions,^24,29^ ensuring that there is no harmful interaction with air or contamination of the sample. However, after annealing at 500 K, all layers of Sb are desorbed except for the w-Sb film, which remains directly bonded to the W(110) surface, as confirmed by LEEM, LEED, and STM experiments. In the final step, Sb was deposited onto the w-Sb/W(110) substrate at 390 K within the STM and ARPES UHV chambers.

The LEEM images, movies, and LEED patterns were recorded during Sb deposition at the specified temperatures. After depositing the α-Sb, the sample was cooled to room temperature for subsequent LEEM and LEED measurements. ARPES spectra were recorded at room temperature following the preparation of the antimonene layer. To achieve atomic resolution, STM measurements were performed at *T* = 4.5 K. STM data were analyzed using the WSXM software.^51^

### Calculations

First-principles DFT calculations were performed using the VASP (Vienna ab initio simulation package)^52,53^ on the GGA-PBE level.^54^ The plane wave basis set was restricted by an energy cutoff of 340 eV. The Brillouin zone was sampled by 6 × 6 × 1 Monkhorst–Pack *k*-points grid.^55^ The total energy convergence criterion was chosen to be 10^–6^ eV between subsequent iteration steps, and the maximum force allowed on each atom during the geometry optimization was less than 0.01 eV/Å. All the atomic positions were relaxed by a conjugate gradient method.

## Supporting Information Available

Movies showing the growth of antimonene at *T* = 390 K, FoV = 10 μm (Movie S1A); the growth of antimonene at *T* = 350 K and *T* = 430 K, FoV = 10 μm (Movie S1B); the growth of antimonene at *T* = 390 K, FoV = 50 μm (Movie S2); the growth of antimonene at *T* = 410 K, FoV = 10 μm (Movie S3); the growth of antimonene at *T* = 330 K, FoV = 50 μm (Movie S4); and the evolution of two-domain structure, *T* = 400 K, FoV = 5 μm (Movie S5) (ZIP)
Results of DFT calculations of band structure of α-antimonene using PBE and HSE06 correlation-exchange functionals (Figure S1); ARPES map presenting constant energy cut at the Fermi level of α-antimonene grown on a w-Sb/W(110) surface (Figure S2) (PDF)
