# Supplementary material for: Epitaxial Growth of Large-Scale α-Phase Antimonene
Source: Nano Lett. 2024 Sep 24;24(40):12469–75. doi: 10.1021/acs.nanolett.4c03277 (PMC11468732; doi:10.1021/acs.nanolett.4c03277)

Supporting Information

Epitaxial growth of large scale α phase antimonene

Tomasz Jaroch, Lucyna Żurawek-Wyczesany, Agnieszka Stępniak-Dybala, Mariusz Krawiec, Marek Kopciuszyński, Piotr Dróżdż, Mariusz Gołębiowski, Ryszard Zdyb*

Institute of Physics, Maria Curie-Sklodowska University, Pl. M. Curie-Sklodowskiej 1, 20-031 Lublin, Poland

Email address: ryszard.zdyb@umcs.pl

All movies present growth of the α phase antimonene on the w-Sb/W(110) substrate.

Movie 1A: Field of View (FoV) = 10 µm, T = 390 K, E = 3.5 eV.

Movie 1B: FoV = 10 µm, T = 350 K (0 – 0.1 ML) plus T = 430 K (0.1 – 1.0 ML). E = 3.5 eV.

Movie 2: FoV = 50 µm, T = 390 K, E = 3.5 eV.

Movie 3: FoV = 10 µm, T = 410 K, E = 3.5 eV.

Movie 4: FoV = 50 µm, T = 330 K, E = 3.5 eV.

Movie 5: FoV = 5 µm, T = 400 K, E = 24 eV. The movie has been recorded using tilted electron beam enabling the observation of antimonene domains.

Figure S1

Results of DFT calculations of band structure of α antimonene using PBE (violet) and HSE06 (orange) correlation-exchange functionals.

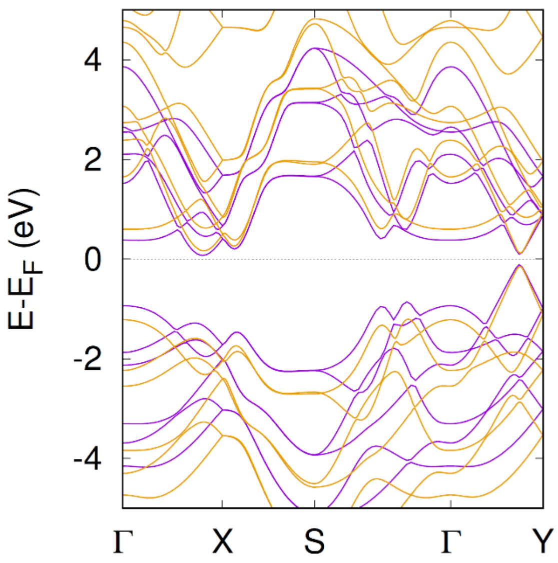

Figure S2

The ARPES map presenting constant energy cut at the Fermi level of α antimonene grown on w-Sb/W(110) surface. The solid white and red lines indicate part of the Brillouin zones of two rotated domains of antimonene. The dotted lines indicate corresponding directions within each Brillouin zone. The rounded rectangular-shaped features located along the $\Gamma\overline{Y}$ directions (one indicated with yellow dotted line) are the cross sections of Dirac cones representing two domains.

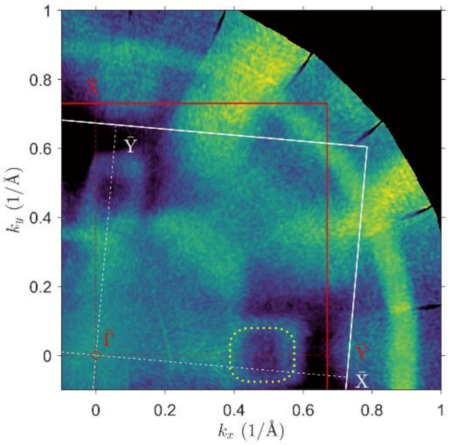

Supplement: Supplementary file 2 — nl4c03277_si_002.pdf [file nl4c03277_si_002.pdf]
